# A Mental Health Storytelling Intervention Using Transmedia to Engage Latinas: Grounded Theory Analysis of Participants’ Perceptions of the Story’s Main Character

**DOI:** 10.2196/10028

**Published:** 2018-05-02

**Authors:** MarySue V Heilemann, Adrienne Martinez, Patricia D Soderlund

**Affiliations:** ^1^ School of Nursing University of California, Los Angeles Los Angeles, CA United States

**Keywords:** depression, anxiety, transmedia storytelling, Internet, cell phone, mental health, eHealth, mood disorders, smartphone

## Abstract

**Background:**

Transmedia storytelling was used to attract English-speaking Latina women with elevated symptoms of depression and anxiety to engage in an intervention that included videos and a webpage with links to symptom management resources. However, a main character for the storyline who was considered dynamic, compelling, and relatable by the target group was needed.

**Objective:**

We conducted interviews with 28 English-speaking Latinas (target group) with elevated symptoms of depression or anxiety who participated in an Internet-accessible transmedia storytelling intervention. The objective of this study was to examine participants’ perceptions of the lead character of the story. Development of this character was informed by deidentified data from previous studies with members of the target group. Critique of the character from a panel of therapists informed editing, as did input from women of the target group.

**Methods:**

All interviews were conducted via telephone, audio-recorded, and transcribed. Data analysis was guided by grounded theory methodology.

**Results:**

Participants embraced the main character, Catalina, related to her as a person with an emotional life and a temporal reality, reported that they learned from her and wanted more episodes that featured her and her life. Grounded theory analysis led to the development of one category (She “just felt so real”: relating to Catalina as a real person with a past, present, and future) with 4 properties. Properties included (1) relating emotionally to Catalina’s vulnerability, (2) recognizing shared experiences, (3) needing to support others while simultaneously lacking self-support, and (4) using Catalina as a springboard for imagining alternative futures. Participants found Catalina’s efforts to pursue mental health treatment to be meaningful and led them to compare themselves to her and consider how they might pursue treatment themselves.

**Conclusions:**

When creating a story-based mental health intervention to be delivered through an app, regardless of type, careful development of the main character is valuable. Theoretical guidance, previous deidentified data from the target group, critique from key stakeholders and members of the target group, and preliminary testing are likely to enhance the main character’s relatability and appropriateness, which can increase sustained engagement.

## Introduction

### Background

Innovative ideas that harness the use of digital storytelling to engage users in mental health interventions involving smartphones, computers, tablets, or other devices merit exploration. Technological apps aimed at early detection, resource use, or Web-based mental health treatment that feature a human character [1,2], embodied conversational agent [3], virtual agent or coach [4,5], avatar, or fantasy character [6] can benefit from attention as to how characters affect user engagement. From Mexico [7] to the United States, photonovelas [8,9], webnovelas [10], and telenovelas to engage Latinos have been used in story-based research on a variety of physical health–related topics [11-16]. The expanding accessibility of the Internet and the growing popularity of smartphones worldwide support the use of story-based media to attract and reach previously unreached populations in terms of mental health or well-being. Many psychiatric outpatients own smartphones; Torous et al [17] found that 200 of the 320 psychiatric outpatients in their study owned a smartphone, and most (70.6%, 141/200) were willing to use their smartphones to run apps to monitor their mental health. Because smartphone use is higher for US Latino adults (aged 18-49 years) than it is for adults of other ethnicities [18], there is great promise for mental health engagement using smartphones with this population. Many US Latinos obtain important health information online. According to an American Trends Panel Survey, Latino adults used smartphones more often than non-Latino whites in 2014 to access online health information; that is, nearly three-fourths (72.94%, 755.5/1035) of Latino adults reported using their smartphones in the last year to access information about a health condition compared with 58.00% (1244.68/2146) of non-Latino whites [19]. Usage of smartphones is also high for individuals living with financial difficulties in the United States; for example, those with a household income of less than US $30,000 per year are more reliant on smartphones to access the Internet (13.00%, 195/1500) compared with individuals earning US $75,000 per year (1.00%, 15/1500) [19].

Recreational use of the Internet is high among US Latinos who speak English (94.0%, 771.7/821) and those who speak Spanish (86.0%, 583.9/679) [20]. Digital and electronic media are important potential avenues for reaching Latinos who may be struggling with untreated mental health issues because of their widespread use. For example, 51.00% (765/1500) of Latino adults sampled by the Pew Research Center reported they played video games on a cell phone or other device [21]. Many video games depict specific characters, but critics complain that few desirable Latino characters are featured in games [22]. When Latina characters are portrayed, they are often depicted stereotypically as maids with heavy accents in provocative clothing [23]. Although electronic games can provide effective, appealing mental health interventions, they must be designed with attention to characteristics of the clients they target, including culture and gender [24]. Attention to problematic depictions of leading characters in electronic games and other media technology apps is crucial to engagement. Historically, entertainment-education media strategies in radio and television broadcast series have involved the use of characters whom audiences could identify with, which led to high viewer ratings and robust social discourse. This has been linked to viewers’ engagement in health promotion activities and positive lifestyle choices, which were influenced by the behavior modeled by characters of the show [25].

### Theoretical Guidance for Development of Characters in Story-Based Media

Theorists have explained that a key element of story-based approaches is how much an audience can relate to the main character. Albert Bandura’s social cognitive theory (SCT) holds that characters who are perceived as interesting or relatable [26,15] are key to engagement with media. A viewer who feels a sense of connection with the main character will have a deeper engagement, which may be enduring over time, leading the viewer to come back for more. For example, in their research with an interactive video drama series aimed at helping men quit smoking, Bottoroff et al [1] found that men who related more strongly to the main character experienced higher levels of support from the series.

SCT [27] purports that communication involves psychosocial mechanisms that influence the thoughts, feelings, and actions of individuals. When communication takes the form of stories dramatized in media, viewers learn vicariously by observing characters as they deal with expected rewards, social consequences, and the influence of values. The story and characters inform viewers in terms of their thoughts and judgments. Due to the power of the story, SCT holds that direct engagement with story-based media can lead to behavior change by informing, motivating, or guiding viewers [26]. Viewers can be further influenced to change their behavior through personal discussions with other people who found the story worth talking about [28]. SCT can be used to guide the development of characters that are featured in any app to enhance the engagement of users. However, creation of a compelling and relatable main character requires thoughtful strategic development with input from the target group of users.

### Transmedia: Storytelling Across Multiple Digital Platforms

Transmedia [29] is a dynamic, Internet-accessible media approach that features compelling characters and uses dramatic storytelling that extends across various digital platforms, including computers, tablets, and smartphones. When designed to engage, entertain, and educate, transmedia productions can be leveraged to attract and sustain the attention of audiences for the purposes of health [30]. Transmedia storytelling attracts users through compelling content and offers users decision-making power to choose when and how they will engage which parts of a story and for how long. This encourages the participant to be dynamically engaged rather than passively entertained. Participants might choose to expand their engagement via story extension videos, bonus videos (featuring a particular character speaking outside of the storyline about related topics), or by using other interactive components of the transmedia package. The participant decides which links to click and which content to explore and does so in a discreet way if they are using their own personal device. The confidentiality of this feature is particularly attractive for smartphone users [31] who might be reluctant, afraid, or unwilling to share anything about their mental health symptoms, their distress, or their interest in mental health treatment resources.

A successful transmedia production to enhance sexual health among teens that has been very popular with Latinos is the Emmy-nominated webisode series, *East Los High*, created by Wise Entertainment, Inc. [32]. Having just completed its fourth season on Hulu, this English-language series featured an all-Latino cast of teen characters attending a fictitious high school in a fictitious community with plots involving romance, humor, sex, friendship, betrayal, success, and loss. The story extended beyond episodes to the show’s companion webpage which had, during its first season alone, 123,728 unique visitors with 870,684 page views from all 50 US states and 163 countries [32]. Although not a prime focus of the show, a trio of story extender videos in the third season explored how one teen character sought support from a therapist as she dealt with past experiences of sexual abuse.

### Objectives

Although the specific focus of how Latina adults deal with mental health symptoms has not been central to any transmedia production to date, US Latino audiences have a high interest in story-based media. According to Nielsen’s Total Audience Report [33], 96% of US Latinos watched television in the second quarter of 2017, 92% engaged apps on a smartphone (including Web-based), and 78% accessed videos on a smartphone. Latinos continue to be over-represented in movie audiences in the US and Canada according to the Motion Picture Association of America [34]; although Latinos are only 18.00% (62,366,197.1/346,478,873) of the population, they accounted for 23.00% (79,690,140.8/346,478,873) of frequent moviegoers in 2016. However, characters depicted in story-based media such as streaming or network television shows or films include relatively few Latinos characters; in 2016, 5.80% (605.7/10,444) of characters with speaking roles were Latinos, and of them, only 37.9% (229.7/605.7) were female characters [35].

Due to the growing population of English-speaking Latinos in the United States and the expansive reach of transmedia via the Internet, our research team created a dramatic story-based transmedia intervention featuring a Latina main character with the aim of attracting and engaging untreated symptomatic Latinas. Informed by SCT, the goal of our overall mixed methods study was to engage viewers to increase their awareness of mental health symptoms, enhance early detection of symptoms, provide an interactive experience to help women contemplate their own situation, inform them of available resources for symptom management, and ultimately increase their behavioral intentions or actions taken to get help. The purpose of this qualitative analysis was to explore, describe, and interpret participants’ perceptions of the main character (Catalina) and to discuss our findings in relation to implications for future development of characters for various technological apps aimed at enhancing mental health or well-being.

### Prior Work

**Figure 1:**
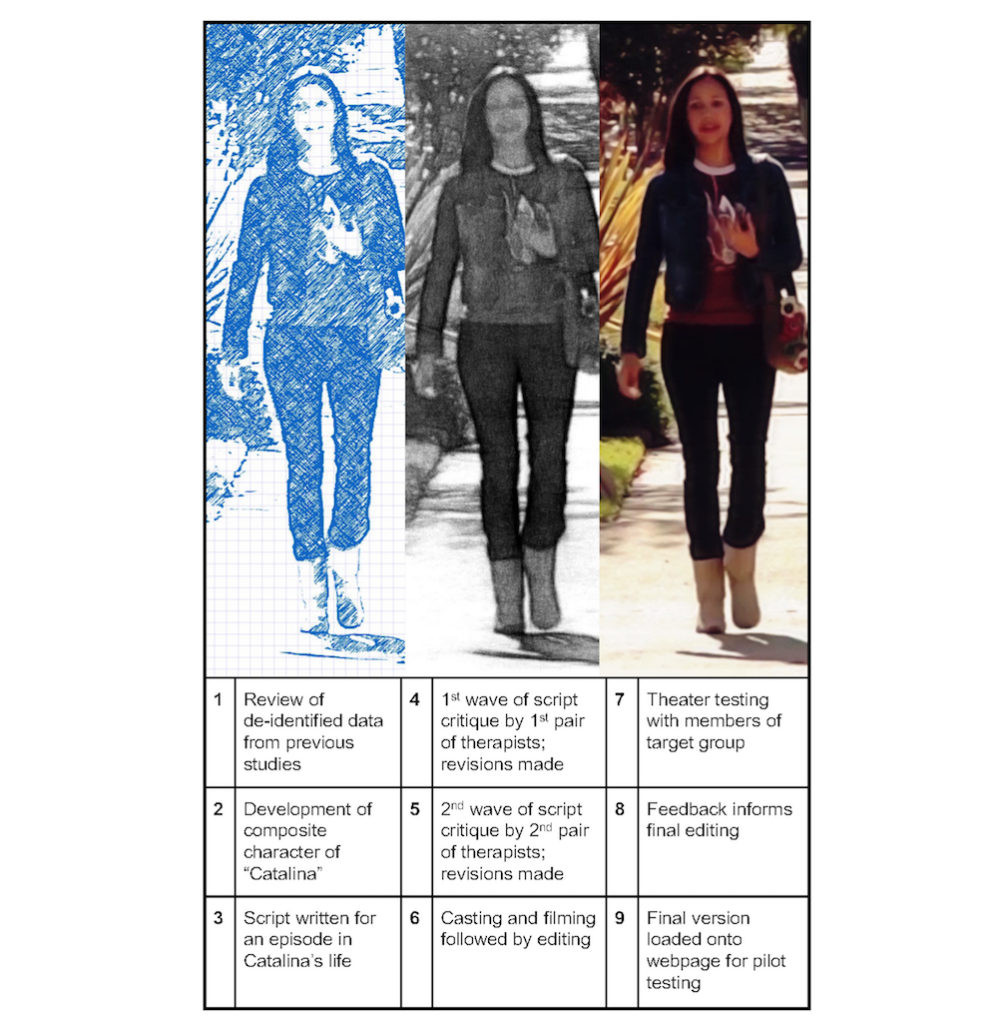
Development of Catalina and her story.

Mindful of SCT [26-28], we invested in a rigorous process for creating our transmedia including the main character, Catalina, which balanced believability with interest, so participants would find it compelling, acceptable, and relevant to their own lives. Under the guidance of the University of California, Los Angeles Institutional Review Board (IRB), a composite sketch for Catalina was developed by the researcher informed by deidentified data from previous community-based, qualitative, or mixed methods studies with English-speaking Latinas of Mexican or Central American descent struggling with depression. From the array of attributes compiled from previous data, the researcher proposed a sketch of the main character, Catalina, including her backstory, life circumstances, concerns, motives, and goals. Catalina was proposed as a 28-year-old Latina who was a single mother of a 4-year-old daughter, living with her parents. The sketch and a basic storyline were presented to a Latino scriptwriter with extensive Hollywood experience who also served as our director and cinematographer; he created the settings, the script, and the trajectory of the drama. A community advisory board, comprising 4 therapists who had significant experience with Latinas in outpatient mental health settings (2 of whom identified as Latinas) read and critiqued the script in 2 waves, paying special attention to the main character. After each wave, the script was revised, and nuanced tailoring was done of the main character to enhance content validity in terms of believability, accuracy, and sociocultural appropriateness. Auditions were held in Hollywood, and Latino actors were hired; filming was done over a 5-day period. After the video scenes were filmed, editing was done. A composer provided a soundtrack. Subsequently, a total of 19 Latina adults from community settings participated in theater testing; feedback was collected via 1:1 interviews and focus groups. This feedback, including input about the portrayal of Catalina, informed the next phase of editing and the final version of the media plus the title, “Catalina: Confronting My Emotions.” Finally, a 3-min introduction video was created. For this, the researcher created the script, one of the character actors did the voice over, and an editor wove together clips from the story to fit the script (see Figure 1).

The story of Catalina is told through events of her everyday life, but a tension develops as the drama unfolds. Over the course of 3 videos, Catalina is portrayed in her own home, on the street in her neighborhood, in a social situation at a party at the home of her best friend, and finally, in a city scene as she leaves a clinic and walks to a bus stop. In each scene, she is either interacting with others or dealing with her thoughts and feelings alone. Through this, Catalina becomes aware that she is experiencing mental health symptoms and grapples with what she should do about it.

The researcher also collaborated with a computer programmer to create a user-friendly project webpage, as was described elsewhere [2]. The goal was to create an interface that had ease of navigation [31], the same aesthetic quality of the videos, plus an attractive, organized display of links on a black background with photos and clear, simple captions so features could be accessed in a sequence. This included links to 3 different videos through which the story of Catalina unfolded (14-, 4-, and 4-min long). The videos were designed to attract the attention of users and enhance their motivation to click the link to watch the next video when the previous one concluded. The 3 videos completed 1 arc in Catalina’s story (1 episode), so there was some resolution at the end. However, to continue with the transmedia intervention, the story-based videos were followed by another set of video logs and an interactive feature involving a character who portrayed Catalina’s nurse therapist, whom we named Veronica Sanchez, RN described elsewhere [2]. The final link on the website led to a blog written from Veronica’s point of view that provided links and contact information for local and online mental health resources including hotlines. An interview was held with participants after engagement with the transmedia. At that time, an additional access code was provided to participants so that they could share the videos, blog, and links with friends and family members of their choice. Results showed that women rated their ability to relate to Catalina at 6.95 on a 10-point scale; approximately 6 weeks after first engaging with Catalina in the media, 75% (21/28) of participants reported they were still thinking about the story-based media, and 79% (22/28) were still talking about it with others [2].

Our in-depth analysis of participants’ perceptions of Catalina was catalyzed, in part, by requests made by participants during postmedia engagement interviews for additional episodes that featured her and her life beyond what was available in the intervention. Some participants expressed hope that there would be future episodes to further elucidate the role played by Catalina’s mother; others wanted to know how Catalina’s romantic life would unfold, what would happen if she pursued higher education, or how her engagement in therapy turned out. Overall, many participants wanted more opportunities to engage future episodes that depicted what might happen to Catalina in the future. In making a direct request for more from the research team, one woman summed up how the story-based videos featuring Catalina made an impact, provided an opportunity for reflection, and motivated her. She said:

...I think it would be great as the study goes if you guys could put more videos out there, more pamphlets, more information on how she [Catalina] actually got out of her situation, because for girls like me that are truly impacted by the videos and it’s a reflection of how you’re feeling, it’s going to be a motivation to see [her] move on with her life. I don’t know if you guys are going to have seminars. I don’t know what the next step [is] with your studies, but it will be great to...have a video to see that she moved on.

In response to such direct requests from participants themselves, we designed this grounded theory (GT) analysis and opened ourselves to gain insight into what made Catalina so compelling to users.

## Methods

### Study Design

Our mixed methods design led to quantitative findings on the feasibility, acceptability, and limited efficacy of the transmedia intervention that have been reported elsewhere [2]. This qualitative analysis was guided by GT methodology to explore, analyze, describe, and interpret interview data on participants’ perceptions of the main character of the transmedia storyline.

### Recruitment and Sampling

After securing IRB approval, purposeful sampling was used to recruit a sample of 28 English-speaking Latina adults with elevated symptoms of depression or anxiety located in a Southern California metropolitan area, described elsewhere [2]. Latinas were eligible if they could read and speak English, were within the age range of 21 to 55 years, had access to the Internet, and met criteria for moderate to severe symptoms of depression (score of 10 or more on the Patient Health Questionnaire-9 [36]) or anxiety (score of 10 or more on the general anxiety disorder-7 [37]). After eligible women provided online consent, completed an online survey (described elsewhere [2]), engaged the media, and were interviewed, they received a US $60.00 gift card via US mail, text message, or email.

### Data Collection

Within 72 hours of viewing the media, individual 1:1 interviews were conducted with participants over the telephone by the third author who is a psychiatric mental health nurse practitioner. Each interview was audiotaped with the participants’ permission and lasted an average of 45 min (range: 29-75 min). Since there was no attrition in this study, all women who participated in the transmedia intervention were interviewed (N=28). By design, each participant was interviewed only once. An interview guide was developed during the planning stages of the study to address a variety of topics; however, participant-initiated divergence from any topic was honored to encourage free sharing about any aspect of their experience with the transmedia or any related topic they found relevant during the interview. After the first participant was interviewed, subsequent interviews with other participants were influenced by earlier interviews, so some questions were added to enhance clarity as the interviewing process progressed. However, the focus of this analysis is limited to women’s perceptions of the main character, Catalina, including what participants thought and felt about Catalina, what they found memorable about her, as well as how and why they did or did not relate to her. After verbatim transcription, all interview transcripts were checked for accuracy and loaded onto Atlas.ti (Qualitative Data Analysis by Scientific Software Development GMbH) [38] to help with data management.

### Analysis Plan

Using the methodology of GT [39,40], initial coding was done using gerunds to focus on the action of the participant. To begin, each line of 10 transcripts was carefully considered and coded. The codes that occurred most frequently or were of particular significance were identified and aggregated into what Charmaz termed focused codes [40]. The focused codes were then used to guide analysis with the rest of the 18 interviews. For this analysis, all coded quotations that invoked women’s perceptions of Catalina were identified, scrutinized, and discussed at length among the 3 researchers. The constant comparison of data with data across all 28 interviews was useful in sorting and analyzing quotations. Simultaneously, coding was done and analytic memos were written so each researcher could ask deeper questions of the data and check for patterns and connections across cases. Reflexive memos helped identify biases that might have been at play because of assumptions of the researchers. All 3 researchers had experience in mental health research and clinical care with Latinas; the second author self-identifies as a Latina. To enhance sensitivity in relation to the processes of coding, dialog among the researchers facilitated recognition of situational and contextual factors for each woman. The sharing of coding processes and memos among the researchers was facilitated by Atlas.ti [38]. Charts were created to organize data and allowed us to more effectively compare codes from all 28 women to develop and test properties within the category. Interactive sessions involving all 3 researchers provided opportunities for debate and discussion of our analytic products (the category and its properties). Moving deeper into analysis, diagrams and drawings were created to clarify dynamics within data.

## Results

### Sample

A detailed report of sampling, demographics, and media viewing habits of the final sample was reported elsewhere [2]. A total of 28 eligible Latinas between the ages of 21 and 48 years provided informed consent, accessed the transmedia website, and completed the entire study.

### Qualitative Analysis

#### One Overarching Category Depicted the Sample’s Perception of Catalina as a Real Person

Knowing that participants expressed a desire for more episodes of Catalina’s life, we moved forward to analyze participants’ perceptions of this character, Catalina, and why they wanted a more prolonged engagement with her and her story. Analyses revealed that participants invoked the character of Catalina as a real person who had a past, present, and future. This led us to use temporality as a heuristic device which allowed a deeper analysis of the data and generated more questions, which is congruent with GT methodology [39]. We explored why participants related to the character of Catalina, how Catalina’s timeline influenced their perceptions of her, and what utility their temporal view of Catalina served as they examined their own life histories, timelines, and circumstances.

**Figure 2:**
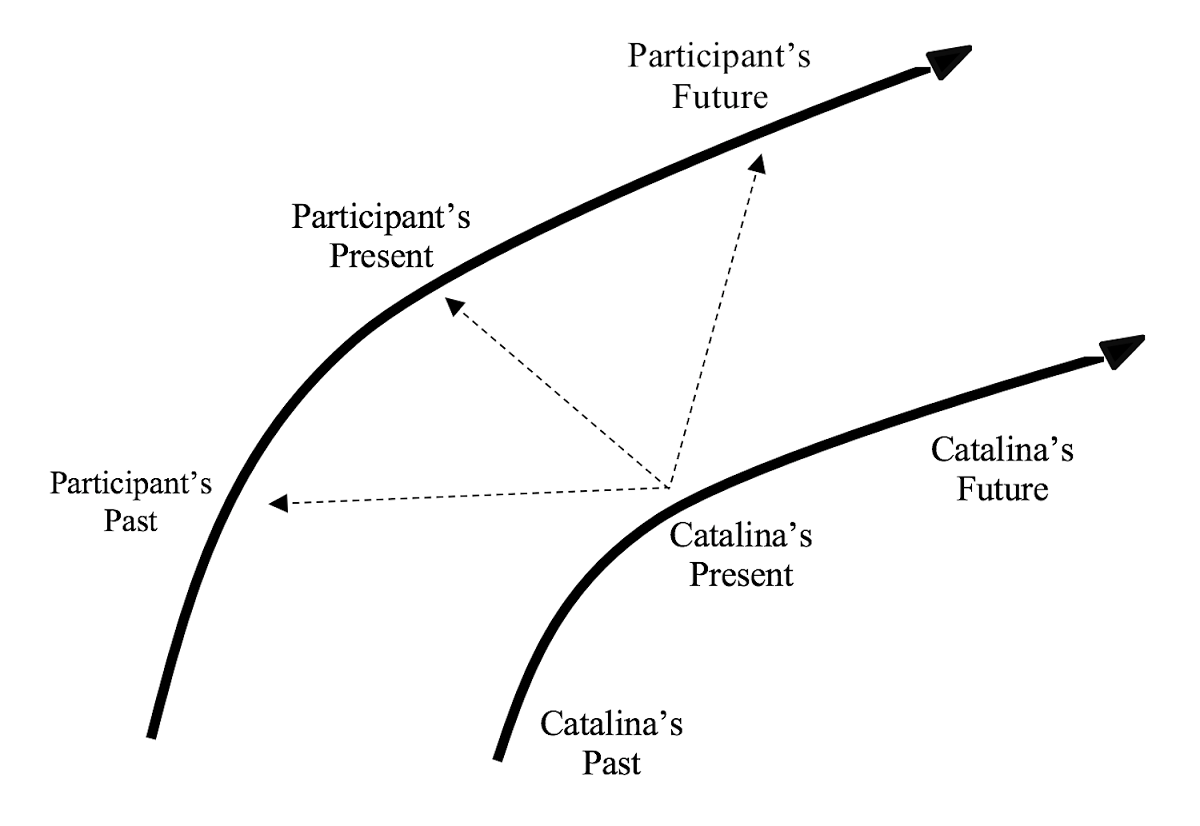
Diagram showcasing one example of how a participant would compare an event on Catalina's timeline with her own past, present, or future.

Our use of the heuristic device of temporality led to the development of the category: She “just felt so real”: Relating to Catalina as a real person with a past, present, and future. We found that while participants were reflecting on a scene about Catalina’s life, it was not uncommon for them to share that it sparked a past memory in a way that brought greater awareness or meaningful insight into their present reality. For some, these memories expanded into thoughts about an imagined future for themselves (see Figure 2).

The women seemed to move easily forward and backward on Catalina’s timeline as they talked about their own. Although the specific details were different for each woman, participants mixed comments about Catalina with comments about themselves as they shared thoughts about their own histories.

#### Four Properties of the Category Revealed How Women Related to Catalina

**Figure 3:**
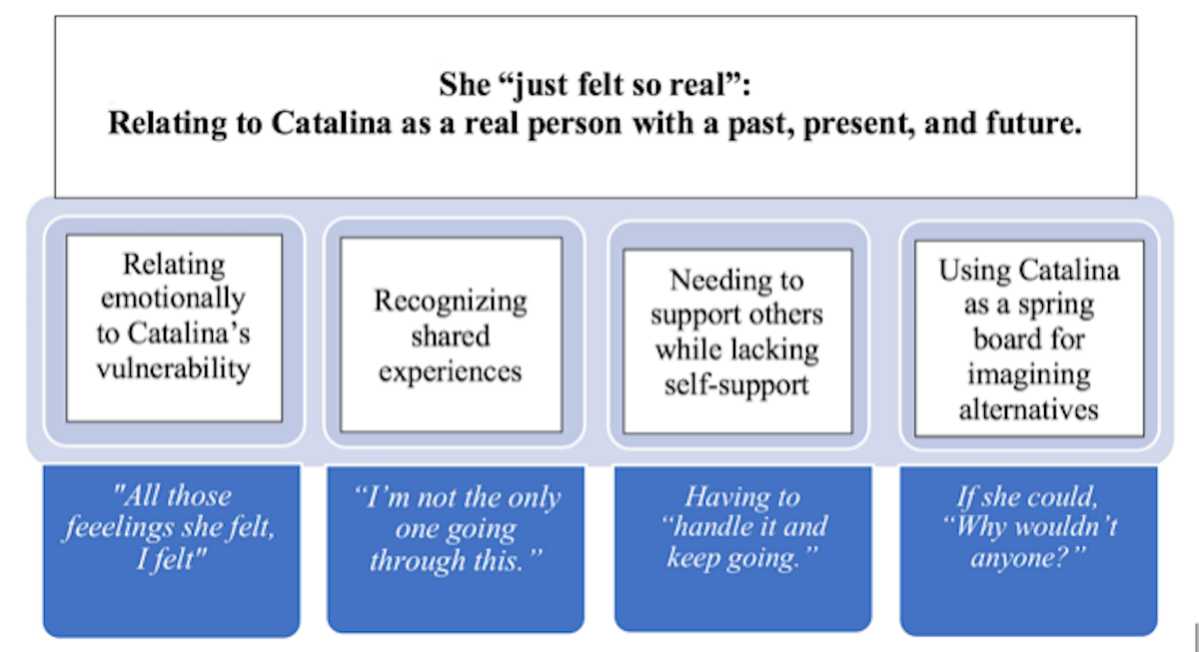
The overarching category (She "just felt so real": Relating to Catalina as a real person with a past, present, and future) and its four properties.

We identified 4 properties of this category, which showed how women related to Catalina. These included (1) relating emotionally to Catalina’s vulnerability, (2) recognizing shared experiences, (3) needing to support others while lacking self-support, and (4) using Catalina as a springboard for imagining alternatives. Each of these properties hinged on the reciprocal assumptions made by the women that, just as they had a past, a present, and an imagined future, so too did Catalina (see Figure 3).

##### Relating Emotionally to Catalina’s Vulnerability: “All Those Feelings That She Felt, I Felt”

When participants started talking about their perceptions of Catalina, their comments easily shifted to a focus on themselves and their own feelings. The women said they related to Catalina and used self-referential phrases, including, “That’s me!” or “I remember that feeling.” Women described how, watching Catalina, they found themselves able to reflect on their emotions although it made them feel vulnerable. One woman admitted:

I know what it feels to be depressed, confused, lost, when you ask yourself, self-doubt[ing], who you really are.

Another connected to Catalina and a feeling of being lost. She explained that she related to a feeling of having “no sense of direction.” Others related to Catalina’s good intentions as someone who was just “trying to survive.”

Several participants had similar emotional reactions to scenes in the drama, but this was not always the case. The same scene did not always stir the exact same emotions in every woman. Nonetheless, the emotional reactions of women connected them to the character. Reflecting on the drama, women seemed to juxtapose Catalina’s emotional reality on their own in the past or the present. One woman stated:

I related to the frustration that [Catalina] felt, to the loneliness, to overwhelmed feelings, a feeling where it’s many things at once. So, overall, a lot of the emotions and feelings that she was feeling in the video, it’s pretty much how I feel right now.

This woman, like many others, used comparisons of herself and Catalina to further articulate details of her current emotional state. Another woman said:

I do feel at times, I feel like I’m going to like, blow up. And, that’s one thing that she did mention about how sometimes she feels like her head is about to explode, and like, I feel the same way sometimes too. Like, sometimes I feel like it’s too much to handle.

Some women related to how Catalina expressed herself emotionally, such as when she cried in one particular scene. After interpreting Catalina’s feelings in that scene, one woman said:

It’s almost like if you sit there and have a pity party for yourself, and that’s not the solution. I mean, I understand what the issue is, I just, the frustration of not knowing how to get out of that.

Speaking about that same scene, another woman extended Catalina’s feelings of vulnerability and helplessness at that moment by expressing her own, exclaiming:

...it’s almost as if there’s nobody out there that can actually help you.

Other emotions spurred by Catalina were linked to feeling “down,” “worthless,” or lacking “confidence.” Women related to what they interpreted as Catalina’s feeling of “low self-esteem.”

##### Recognizing Shared Experiences: “I’m Not the Only One Going Through This”

In addition to feelings, women related to the situational predicaments of Catalina’s life. Specific aspects of Catalina’s struggles at various time points throughout her story resonated with participants’ struggles. This enhanced their ability to connect. Women said they realized that if Catalina was experiencing this, then they were not alone. One woman said:

I’m not the only one going through this.

Another woman summed it up saying she related not only to the demographic and emotional situation of Catalina but also the way she engaged in negative self-talk:

I related to a lot of the parts—like the child, the going out part, the being sad part about a guy—what else?...And then, she was just putting herself down.

Women recalled particular instances when they had grappled with specific problems and how their struggle felt similar to Catalina’s struggle. For example, one woman related to Catalina’s disappointment with a romantic partner saying:

It just made me really sad, because I know how that feels when you get ditched by somebody. I just felt bad for her, even while she was just like a character in the movie, [she] just felt so real. Like, I can relate to her.

Women spoke about how their past impacted their present situations. For some, this included memories of a past event or situation that was troubling them now. Some women compared themselves and pointed out that they had made better decisions than Catalina did, for example, in romantic relationships. Others felt their experiences were worse than Catalina’s. For example, thinking about the story, one participant identified with Catalina’s decision to accept a ride home from a man whom she just met at a party. She shared a memory when something similar but “worse” had happened to her at a young age, a traumatic incident from her past. However, rather than discouraging her from continuing to engage the media, the scene encouraged a deeper connection with Catalina. She found the scene to be “really, really scary,” but it led her to relate and then confide how this negative past experience “made” her “extra cautious” from that point on in her life, shaping the way she looked at the dangers of social encounters.

Various women related to Catalina’s demographic situation as a single, working mother who was going to school. One woman emphasized how, like Catalina, she was “still” living with her parents. She likened her experience to Catalina’s saying:

I feel like I’m stuck as well.

Other participants described a connection with Catalina despite demographic differences. One woman who self-identified as a lesbian said she identified with Catalina although, unlike her, the character was involved romantically with a man and had a child. She said:

It would be nice to feel like I would identify with her if she were gay or anything, but that’s okay. I can still identify or relate [to her] on another note, in another, you know, whatever [an]other aspect of her life...I did feel as though I’ve been in her shoes before. I’ve never been a mother and I’m not a heterosexual woman. Actually, I identify as lesbian [but] I do have a working class family and I did reside at home with my family before moving in with my partner.

Although married, another woman who said that she had a supportive husband related to Catalina’s sense of being alone. She said:

[He] just doesn’t understand me, like how Catalina would want people to understand her.

Many women found a connection because Catalina seemed to be asking herself deeper questions about which direction she should take in life. One woman related to Catalina’s experience of not knowing what to do, saying:

It doesn’t have to be that I’m a single parent or whatever. Just whatever life throws at you, at times, we don’t really know how to deal with it.

##### Needing to Support Others While Lacking Self-Support: Having to “Handle It and Keep Going”

Participants related to Catalina as a mother, daughter, or a partner who needed to support others even if she did not have much support for herself. Several women felt close to a breaking point which they also saw in Catalina. Women recounted how “stressed” and “overwhelmed” they felt in their present situation, how they lacked sufficient support to carry the load, and how it seemed that there was no end in sight for their struggle. To them, their troubles seemed to be ongoing into the future without a viable resolution. Women disclosed how they felt responsible in their role as a parent and tended to ignore their own needs by deciding to “handle it and keep going.” Recalling a scene when Catalina was talking about her role as a mom, one woman said:

[Catalina] saw her baby kind of lying there as she slept, and she’s kind of like freaking out inside. I can relate to that. Even though I’m the adult, I don’t know the answer to everything, but I have to keep it together because someone else depends on me.

Women related to how diligently Catalina “struggled” to hold things together for herself and her family. One woman said:

She’s just trying to succeed and do better for her kid.

Another woman confided how she felt compelled to work weekends so she could “pay all the bills” so she related to how “hard” it was for Catalina to be a single mother; she too lacked time to spend with her children or to “do stuff for yourself.”

Women explained that, just like Catalina, the pressure to stay solvent financially brought stress that eroded confidence in themselves and their parenting abilities. One woman who lived with her spouse disclosed that she felt:

...overwhelmed with money situations and trying to survive and knowing if I’m a good Mom.

Another contrasted herself with Catalina and explained her belief that she had to make sacrifices when she became a parent, for better or worse, which eliminated options and reduced her freedom. She explained that as a single mother, she had not gone out “for fun” since her daughter was born 3 years ago, but she reasoned:

...that’s just the things that you give up, in my opinion, [for] kids.

After watching Catalina interact with her family in the story, some women admitted how their efforts to support their family had negatively impacted their lives. They acknowledged that, like Catalina, they wanted to have more support. They found themselves wishing they “could talk to someone” about their problems or that they could meet a romantic partner who would be supportive. But, many women felt that there was no way to actually meet their needs. They expected themselves to be able to handle all the responsibilities of motherhood on their own. A participant who was a single, working mother and student reluctantly admitted just how difficult her day-to-day life had become and how she was trying her best to hold herself together. She felt disillusionment in her struggle to persevere in her situation but still tried to be optimistic. Echoing the words that Catalina spoke in the story, she said:

I mean, everything’s going good, but I do feel at times, like I’m going to like, blow up.

Just how long participants would be able to carry on without sufficient support was left as an open question for many women.

##### Imagining Alternatives Through Catalina: If She Could, “Why Wouldn’t Anyone?”

Participants related to Catalina and used comparison to imagine a future situation for themselves. When they perceived Catalina as navigating an experience they had not yet encountered, they contemplated this as a future possibility in their own lives. This led them to reflect on their past and, in tandem, to project into their future. Positive aspects of Catalina’s story motivated some women, whereas others were catalyzed by negative aspects. For example, scenes that depicted Catalina before she got help for her emotions led some women to realize how much they did not want to live like that in the future. One woman said:

Seeing how the video shows that since her baby was an infant, I think she was just a newborn when [Catalina] split with her partner, and me seeing that years had gone by [in a subsequent scene], because the little girl was bigger and [Catalina’s] still trying to get out of her situation and she's still depending on that father to provide for her financially, it made me see that this is not how I want to be. I don’t want years to go by in the same situation. I don’t want that. Yeah, I don’t want that.

Another woman used the video instrumentally. She thought about what she saw happening to Catalina, and this “pushed” her toward taking action. She said:

I was planning on getting [help] anyways, but once I saw the video it pushed me to not wait on it. I used the video to kind of help me on like okay, you can't wait on therapy because if you do, it can prolong. And as time goes on, and like Catalina seemed so much down, I think it's not healthy emotionally, physically, and mentally. So, the video did give me a little bit of a push to do it now instead of waiting and see where I can go, you know?

Other women focused on what they perceived as positive in that Catalina acted on her own behalf to get help. Women talked about the scene in the story when Catalina was coming out of a neighborhood clinic where she had engaged in therapy sessions with Veronica, another Latina character who was a nurse therapist. They compared this situation with their own and reflected on choices they could have made in the past or might make in the future to get help for themselves. Several women perceived Catalina to have greater self-awareness and more positive feelings at this point in the story. They saw her as having more self-esteem, as carrying herself with more confidence. They proceeded to imagine how, like Catalina, they could adjust their own paths moving forward. One woman said she learned helpful lessons from Catalina:

Catalina was teaching me how to start getting help and how to go get help and understand myself. Then after, it seems like, after the first session she was feeling much better about herself, and she enjoyed it [therapy].

Through the scenes of Catalina’s life, whether focusing on the negative or the positive, participants started envisioning a future strategy for themselves. One woman focused on Catalina’s courage to get help and surmised:

Just seeing that, like, you know, the kind of life that she had, if she was able to find time and make it possible then, like, why wouldn’t anyone?

Others imagined seeking help but recognized personal fears about seeing a therapist. They related but looked past the fear to see the bigger picture. One woman said:

Like, towards the end of the video, you can tell her personality was changed. She seemed happy. And she was scared to see a therapist, like myself, and that was actually a positive outcome.

Seeing Catalina as a Latina who overcame barriers to get help, one participant articulated her perspective about cultural expectations in her community, feelings of shame about needing mental health care, and the pressure to hide emotional issues. She said:

It's very difficult, [in] the Hispanic culture, to get help, because Hispanic women keep it to themselves for a very long time. And they feel ashamed. But on the video, it makes you just want to go get help because of the situation Catalina was having. Because, you know, sometimes you say to yourself, “No, this cannot happen to me. No, this will never happen to me.” But, in reality, it is happening to you.

## Discussion

### Principal Findings

Having received requests from Latinas in our sample for more videos featuring the main character of our story-based transmedia intervention, we did a GT analysis of participants’ perceptions of the main character (Catalina) and found that they related to her socially and emotionally. Participants found Catalina’s life and story to be familiar and realistic to the contexts and situations of their own lives and those of their friends or family. As discussed by Bandura [26], Catalina was relatable, compelling, and likable as a Latina who dealt with her situation by getting help. Our participants related to Catalina’s emotional experiences and variously defended her, critiqued her, or supported her actions as reasonable in her situation. Although we did not ask how entertained women were by the story, none of the women dropped out of the 6-week study after watching the introduction video, and all women chose to click to watch all 3 story-based videos of Catalina’s story [2]. This implies that women were engaged in the transmedia intervention based on Calvo and Peters’ [41] definition of engagement as a “combination of motivated commitment and sustained attention.” During interviews, women spoke openly about Catalina, often relating to her emotional vulnerability and the ways she supported others even when she did not have the support she needed. An indication of the impact Catalina had on participants is that most (75%, 21/28) of the sample were still thinking and talking (79%, 22/28) about Catalina’s story and the transmedia 6 weeks after first engaging with it [2].

The sample’s engagement with the intervention seems to have been enhanced not only by their ability to relate to Catalina but by a dynamic of comparison that the production spawned. The women actively compared themselves with Catalina through a process that involved reflection and imagination, and they accomplished this with the help of temporality. Because they perceived Catalina to be like a real person with a past, present, and future, they used temporality in a fluid way to traverse her timeline and then reflect on or imagine themselves at various points in their own lives. Because of Catalina, they transported themselves to different time points in their own lives, remembering some of their own life events, reviewing their emotions, and comparing their own experiences with hers. Participants tracked the events of their lives and made sense of them while using the Catalina character as a benchmark. This allowed them to move beyond her story to focus on their own. They engaged in a dynamic reconsideration of the past, a focus on the present, or a sizing up of future possibilities.

In addition to relating to and comparing themselves to Catalina, participants seemed to take the next step described by Bandura [26] and learned from her. It is likely that various aspects of the character contributed to this phenomenon such as who she was from their point of view, how she acted and reacted in situations, and what she shared about herself and her feelings throughout the story. Just as Botoroff et al [1] found with their smoking cessation–oriented video drama series, it was the challenges of the main character that participants seemed to find the most compelling. The portrayal of both successes and failures in Catalina’s life may have deepened women’s ability to relate to her and to learn vicariously as they imagined alternatives for their own lives, as proposed with SCT [27,26]. The person women perceived Catalina to be in the past, present, or future and how she reacted at different time points, all seemed to serve as grist for learning about themselves as they compared and contrasted her life with their own. Through multiple connections and comparison points, participants could consider possible outcomes they desired, hoped for, feared, or wished to avoid.

By watching Catalina, some women voiced a newfound acceptance of their own experiences as normal. Some identified with Catalina’s feelings of being overwhelmed to the point of exasperation, and others related to her disappointment in not being successful at navigating a romantic relationship. Ultimately, the women in our study found Catalina to be relatable as a person who, just like them, sometimes failed to make good decisions, and at other times took courageous steps.

When focusing on the future, Catalina served as a catalyst for women to think about possibilities for seeking help to enhance their emotional health. Engagement with Catalina and her story supported their ability to consider alternate decisional pathways they had not used before. They were able to contemplate certain actions they could take in the future by considering actions they saw Catalina take in the videos. They wondered if these could be a possible direction for them. For many, using Catalina as a springboard added a dynamic of hope or inspiration.

In the present, women across our sample identified specifically with how Catalina reasoned and responded to life experiences and how she conveyed emotions. By embracing her as an actual person, Catalina’s ongoing timeline seemed to become a vehicle for a deeper connection to an inner life they recognized and valued in the present moment.

With a focus on Catalina’s past decisions and predicaments, some women simultaneously projected themselves into the role and were able to identify patterns, make connections, or learn from past events in their own histories. This gave some insight they previously had not appreciated and brought a different way of thinking about their own life course and past circumstances. Observing Catalina’s life history and past behaviors had the effect of allowing the women to contextualize the factors that set up their own situations and recall how they had reacted emotionally as a result. This identification with Catalina’s past allowed some of the women to be more accepting of their past feelings and emotional reactions.

Some women saw Catalina as stronger and more insightful after she sought therapy, as depicted in the last story scene. Some expressed positive thoughts about this ending scene, reported feeling inspired, and linked this to a pivot in their willingness to pursue the option of therapy for themselves. This highlights the importance of what Calvo and Peters identified as the peak-end rule [41]—that is, of all aspects of their experience with our transmedia intervention, participants may have remembered most vividly both the peak of their experience with Catalina and also their feelings at the end of the media experience. Thus, as app designers, we were wise to pay careful attention to how we presented Catalina within the most exciting or gripping parts of story and also how we showcased her in the last scene of the story when she was talking about her feelings after having gone to therapy, a scene that many women found memorable. It is possible that participants’ ability to relate to Catalina and desire for her storyline to continue beyond the intervention’s bounds, was in part due to the women’s perceptions at these key moments and what they ultimately remembered about her.

Overall, the character of Catalina seemed not only to represent who the women were but potentially who they might become. Connection to the character via the intervention seemed to represent a viable and important mechanism for them to continue to think and talk about her and potentially to learn more about themselves and their options.

### Implications

A rigorous and layered understanding of how and why women related to Catalina provides guidance for researchers planning to create characters for story-based technology apps and interventions. The time we devoted to a solid methodological approach to developing Catalina was crucial for maximizing the power of the character; this included creating a composite based on previously collected, deidentified data from Latinas from the target group, allowing multiple phases of script critique from key stakeholders such as community-based therapists who were very familiar with the target group, theater testing with members of the target group to identify Latinas’ reactions to her, and editing before pilot testing. As Singhal emphasized, relatable characters are more entertaining and can serve as valuable role models by positively influencing viewers’ lifestyle choices and health promotion activities [25].

Commitment to the ethical protection of study participants who provide data to be used for character development is of high importance when creating media for interventions. The goal is to create characters, settings, storylines, plots, and scripts that participants will find relatable, culturally appropriate, and compelling but not offensive or demeaning. Thus, it is best for the research team to include members of the target group and their advocates, so their input is inherent in the development of the narrative. Active consultation with the IRB will help maximize appropriateness of characters and storylines.

Future research can build on this work. A randomized controlled trial is needed to test the effectiveness of our story-based videos compared with videos that are psychoeducational only, without characters. Another valuable study would be to share the transmedia program featuring the character of Catalina with women of other ethnic groups and investigate how relatable the character was to them and if it had an impact on confidence, help-seeking intentions, or action taken. In addition, the transmedia could be shared with men who are struggling with mental health symptoms themselves to determine the impact on help seeking or the impact on their actions to help other women in their lives who are struggling with untreated depression or anxiety symptoms.

### Limitations

This transmedia intervention involved multiple videos but was limited to one story arc. Also, access to the transmedia webpage was tracked only for 6 weeks with this feasibility study. Since we used purposive sampling with a flier that described the study as involving the use of a smartphone, tablet, or computer, self-selection of participants may have occurred. Women who were already interested in and felt competent with technology may have been especially attracted to the study. Their past experience could have made their use of the technology more comfortable and may have positively impacted their experience with the transmedia overall or the characters in particular.

### Conclusions

Stories with a compelling, desirable, relatable, socially, and culturally appropriate main character offers a powerful strategy for sustaining user adherence and engagement in a variety of technology apps that aim to enhance the mental health and well-being of various populations. Theory-based designs, data-informed characters, and culturally appropriate stories that are compelling to the target group are possible if collaborations include not only technology and mental health experts but also stakeholders and members of the target group. More creative experimentation holds abundant promise for deepening and extending user engagement in technology apps to enhance mental health.

## Abbreviations

GT: grounded theory
IRB: institutional review board
SCT: social cognitive theory
